# Evaluation of a planar diode matrix for SRS patient‐specific QA in comparison with GAFchromic films

**DOI:** 10.1002/acm2.13947

**Published:** 2023-07-05

**Authors:** Erminia Infusino, Anna Ianiro, Stefano Luppino, Sandro Nocentini, Cristina Pugliatti, Antonella Soriani

**Affiliations:** ^1^ Department of Medical Physics IRCCS Regina Elena National Cancer Institute ‐ Rome Italy; ^2^ Department of Medical Physics Michele e Pietro Ferrero Hospital Verduno Italy

**Keywords:** Cyberknife, pre‐treatment verification, quality assurance, radiosurgery

## Abstract

**Purpose:**

We validate the routine use of a two‐dimensional (2D) diode matrix for patient specific pre‐treatment verification for Cyberknife (CK) stereotactic radiosurgery and to compare it with film dosimetry.

**Materials and method:**

A total of 46 patients were selected according to the most frequent diseases treated at our institution with the CK system, that is, brain metastases, meningiomas, spine metastases, and prostate tumors. All cases were evaluated with GAFChromic EBT‐3 films and SRS MapCHECK for Fixed cone, IRIS, and MLC collimators of the CK.

**Results:**

The highest mean passing rate was observed for the SRS MapCHECK system compared to films. In order to assess if the two techniques provide statistically different results, a Wilcoxon Signed‐Rank non‐parametric test was performed (*p* < 0.05) and we found gamma values significantly lower for EBT‐3 films with respect to the SRS MapCHECK. We noticed a moderately significant association between the two techniques using Spearman's rank correlation coefficient (*rs* > 0.4). We also performed the Bland–Altman statistical method: less than 5% of the differences resulted outside the range (mean ± 1.96 × SD), so the two methods can be considered interchangeable within the combined inaccuracy.

**Conclusions:**

The use of SRS MapCHECK for CK patient specific quality assurance (QA) is feasible for a variety of clinical districts and could be reliably used as a replacement for radiochromic films.

## 1 INTRODUCTION

Stereotactic body radiotherapy (SBRT) and radiosurgery (SRS) treatments allow the delivery of high conformal dose distributions in 1 to 5 fractions, providing a high biological effective dose (BED). One of the characteristics of SBRT/SRS treatment planning is the use of smaller target margins, in order to minimize the dose to the surrounding normal tissues, accounting for tumor motion and setup reproducibility.^1^

The CyberKnife (CK) device (Accuray Incorporated, Sunnyvale, USA) is a robotic system specifically developed for SRS/SBRT treatments, being able to deliver highly irregular and steep dose gradient distributions using several non‐isocentric and non‐coplanar beams of various sizes.^2^ The system tracks the target position with sub‐millimeter accuracy at regular intervals, assuring the employment of the smallest target margin possible. The complexity of a CK plan could be much greater than intensity modulated treatment plans delivered with conventional linear accelerators, requiring a higher number of monitor units (MUs) and longer treatment times.

Patient‐specific quality assurance (QA) is a crucial step in the overall quality control of radiation treatments in general, as it assesses that the delivered dose accurately matches the planned dose distribution. The high complexity of CK plans requires a strict comprehensive QA for accurate treatment delivery.^3^

For routine patient‐specific QA and end‐to‐end tests, radiochromic films are currently employed.^4^, ^5^, ^6^ In general, film dosimetry is a well‐established method for verifying SBRT dose distributions with small fields due to its optimal spatial resolution.^7^

A QA plan is delivered, measured, and compared with the dose calculated at the treatment planning system (TPS) using a gamma‐index pass criteria, assessing both spatial and dosimetric accuracy of delivery. However, meticulous and time‐consuming film calibration and readout protocols are required.^8^ Moreover, film readout is delayed in time so the treatment workflow requires well‐planned scheduling.

At present, unlike other complex treatment modalities as IMRT and VMAT, patient‐specific QA recommendations for CK are very limited. For example, AAPM‐TG‐135^3^ recommendations mainly focus on machine specific QA. The conventional method for patient‐specific QA of measuring a point dose using an ionization chamber or of measuring a fluence using a standard two‐dimensional (2D) array is inadequate for highly modulated treatment fields with sharp dose gradient fall‐offs that involve small volumes.^9^, ^10^, ^11^

Radiochromic film measurement is the current method of choice both for routine QA as well as specific validation of patient treatment plans for the CK.^3^, ^12^ The commissioning and validation for film‐based CK DQA using various test scenarios^12^ was recently reported and results confirmed that, by means of accurate film‐to‐plan registration, maximum Gamma‐Index pass‐rate search and tight distance‐to‐agreement (DTA) criteria, small errors in beam delivery and system miscalibration can be detected.’’

SRS MapCHECK (Sun Nuclear Corp., Melbourne, USA) is a 2D diode matrix specifically designed for SRS plans and supports both conventional linacs and CK systems. It will eliminate the delay of film processing and will improve QA confidence compared to the use of a single ion chamber or conventional 2D arrays, thanks to the 1013 diode dose measurements performed.

The purpose of this study was to validate the routine use of SRS MapCHECK for patient specific pre‐treatment verification for a variety of CK treatments and to compare it with film dosimetry.

We also studied the correlation between the gamma passing rate and the target volume.

## 2 MATERIAL AND METHODS

### 2.1 Patients selection

In order to validate the clinical use of SRS MapCHECK, we used a data set of 46 patients with different pathologies, who were treated at our Institution with the CK device between February 2019 and March 2020. Table 1 resumes the main characteristics of the cases that were selected.

**TABLE 1:** Main characteristics of the cases selected in our study.

Treatment site	Number of patients	Prescription dose (cGy)	Number of fractions	Collimator
Brain metastases	10	2100	1	Fixed
Brain meningioma	13	500	5	Iris
Spine metastases	13	1200	2	MLC
Prostate	10	950	4	MLC

All patients underwent simulation using a GE Lightspeed CT scanner (GE Healthcare, Boston, USA) with a 1.25 mm step.^13^, ^14^

Radiation oncologists used Eclipse TPS (Varian Medical Systems, Inc., Palo Alto, USA) version 15.6 to contour PTV and OARs, following standard contouring protocols.

### 2.2 Cyberknife device

CyberKnife–M6 (Accuray Incorporated) is a robotic radiosurgery image‐guided device consisting of a compact linear accelerator mounted on an industrial robot with a 6‐axis manipulator arm, whose range of motion is restricted to a hemisphere around the patient.^15^, ^16^, ^17^

The beam source is a 9.3 GHz x‐band accelerator producing 6 MV flattening filter free (FFF) x‐rays from a tungsten alloy target at a fixed dose rate of 1000 MU/min.

Different secondary collimator configurations are available:

1. Twelve fixed collimators, with circular apertures with a diameter ranging from 5 to 60 mm defined at a source‐to‐axis distance (SAD) of 800 mm.
2. IRIS collimator, composed of two stacked hexagonal banks of tungsten segments that together produce a 12‐sided aperture with the shape of a regular dodecagon. The aperture is adjustable under computer control and provides the same 12 apertures as the fixed collimators.
3. InCise Multileaf collimator (MLC), with two banks of 41 tungsten leaves 2.5 mm wide to create shapes as large as 100 × 97.5 mm^2^ at 800 mm SAD. All leaves have full interdigitation and overtravel. The MLC aperture is adjustable under computer control and can deliver variable shaped beams from each LINAC position.

CK allows the delivery of non‐coplanar nonisocentric treatment beams and ensures a sub‐millimeter accuracy by tracking the position of the target with two orthogonal integrated x‐ray imaging systems that provide image guidance for the treatment process. The x‐ray images are registered with reference DRRs reconstructed from the planning CT, on the basis of the location of fiducials. The machine then corrects the positions of the beams according to the resulting shifts.^3^, ^18^

### 2.3 SRS MapCHECK detector and StereoPHAN phantom

SRS MapCHECK and StereoPHAN (Sun Nuclear Corp.) represent innovative solutions for stereotactic end‐to‐end QAs optimized for CK applications. They are compliant with the requirements of the American Association of Physicists in Medicine (AAPM) Task Groups 101^1^ and 218.^19^

The SRS MapCHECK is a high‐density solid‐state diode array. The 1013 SunPoint n‐type diodes cover an active area of 77 × 77 mm^2^. They have sub‐millimetric resolution (0.48 × 0.48 mm^2^ area and 0.007 mm^3^ volume) and high sensitivity, around 15 nC/Gy. The diagonal physical distance between two diodes centers is 2.47 mm.^20^, ^21^

A polymethyl methacrylate (PMMA) packaging (320 × 105 × 45 mm^3^) houses the diodes array. It includes also four fiducial markers for detector alignment and tracking by kV and MV images, strategically positioned for optimal IGRTs with CK.

**FIGURE 1:**
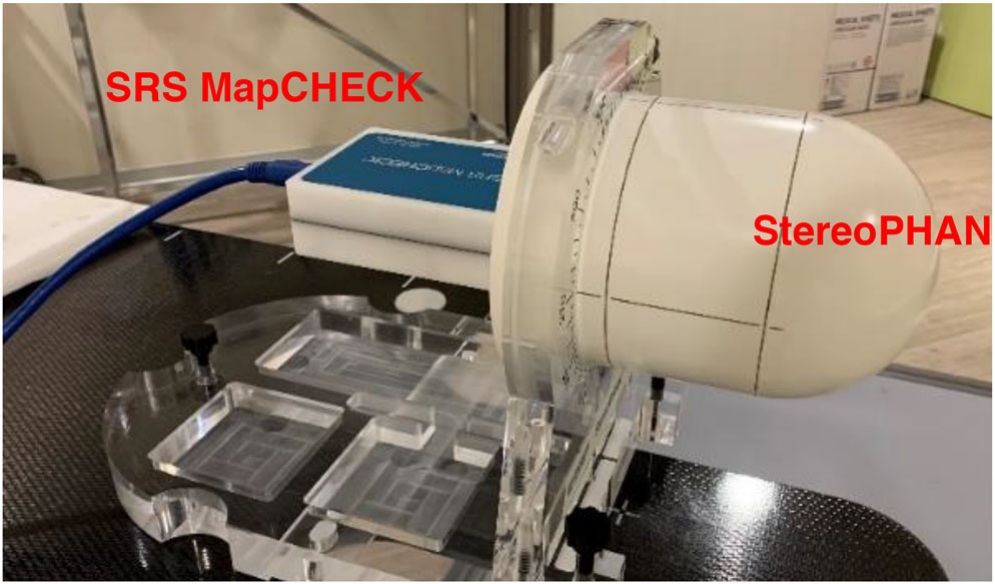
The SRS MapCHECK detector assembled with the StereoPHAN PMMA phantom. PMMA, polymethyl methacrylate; SRS, stereotactic radiosurgery.

The StereoPHAN is a PMMA head‐shaped phantom designed to accommodate different cubic inserts (85 × 85 × 85 mm^3^) for end‐to‐end quality controls (dosimetric QA, images fusion, geometric and optical isocenter verification, etc.) in addition with SRS MapCHECK (Figure 1).

**FIGURE 2:**
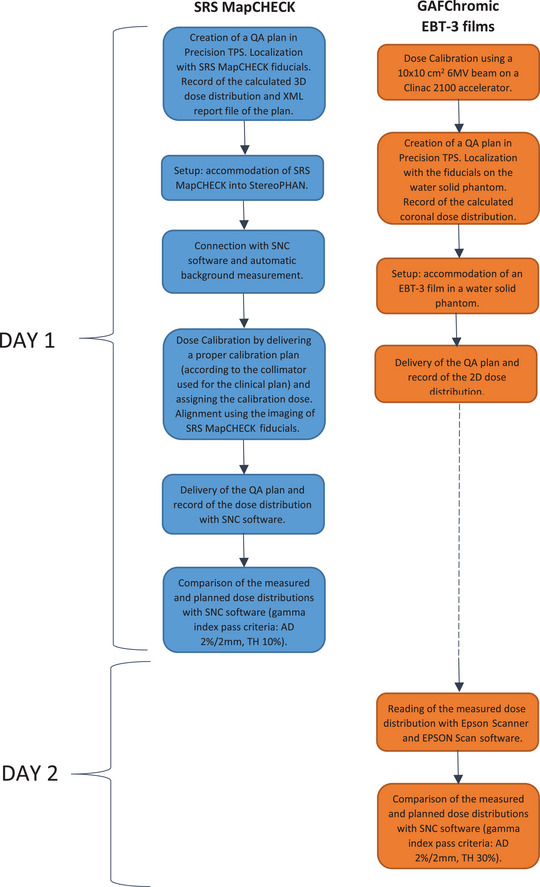
SRS MapCHECK (left) and EBT‐3 film (right) patient QA verification workflows. QA, quality assurance; SRS, stereotactic radiosurgery.

It is possible to lock the StereoPHAN to the TDD treatment couch using an indexing bar, in order to allow the inserts replacement maintaining the initial position and preventing any undesired movement (Figure 2).

**FIGURE 3:**
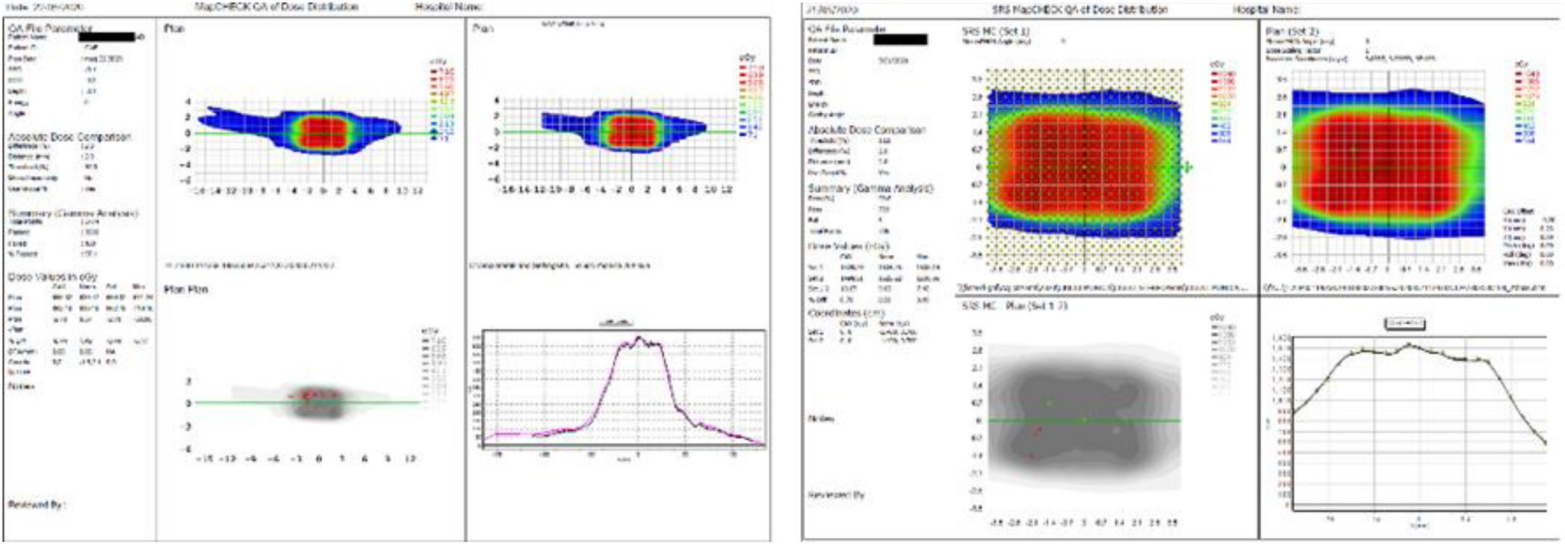
GAFChromic EBT‐3 film (left) SRS MapCHECK (right) gamma analysis report for a representative spine patient. SRS, stereotactic radiosurgery.

The detector interfaces with the SNC software version 8.3 (Sun Nuclear Corp.) that performs automatic background measurement and allows daily dose calibration for each collimator, accounting for output fluctuations and avoiding pressure and temperature corrections (Figure 3).^22^, ^23^

### 2.4 Array and dose calibration

Two calibration measurements are needed before DQA with SRS MapCHECK: array and dose calibration.

Array calibration is necessary to determine differences between SRS MapCHECK detectors and introduce a correction factor for each of them. The SRS MapCHECK full array calibration procedure involves multiple shifts and rotations of the device with respect to the radiation field. A first set of measurement was acquired with a Varian 2100C/D accelerator. Four measurements were performed positioning the array without the StereoPHAN directly on the treatment couch at the beam isocenter, using a 10 × 10 cm^2^ AP field (6 MV WFF, 200 MU). Other four measurements were performed with a 10 × 10 cm^2^ PA field.

A second set of measurement was performed at the CK device. Two measurements were performed with a 5.4 × 5.4 cm^2^ MLC field (6 MV FFF, 200 MU), inserting the SRS MapCHECK in the StereoPHAN: one measurement was recorded with the array in AP position and the other was performed flipping the array in PA position. After all measurements, the relative sensitivity of all detectors was established and the information required for diode angular correction obtained.

Absolute Dose Calibration was needed for each delivery modality (fixed cone, Iris, and MLC). CT scans of SRS MapCHECK inside the StereoPHAN were acquired. The CT was imported into Precision TPS, and the entire phantom volume was overridden to 1.2 g/cm^3^ mass density. For fixed cone and Iris collimator, an isocentric plan with a single QA beam was generated with a 60 mm fixed cone or a 60 mm Iris aperture, respectively, centered in correspondence of the array central detector. For MLC, a sequential optimization plan was generated with a single QA square beam (5.4 × 5.4 cm^2^) centered in correspondence of the array central detector. All plans were normalized prescribing a mean dose of 200 cGy at the central detector. These plans were then used for absolute dose calibration, irradiating the SRS MapCHECK installed inside the StereoPHAN.

Other calibration factors were provided by Sun Nuclear, including angular, dose rate, field size, and temperature correction factors. The effects of these factors on measurement results were well studied elsewhere.^23^, ^24^, ^25^

### 2.5 GAFChromic EBT‐3 film detectors

GAFChromic EBT‐3 films (Ashland Advanced Materials, Bridgewater USA) have a dynamic range designed for best performance in the dose range 0.2–10.0 Gy, making it suitable also for CK applications. The film active layer (28 μm nominal thickness) is located between two 125 μm matte‐polyester substrates and contains the active component, a marker dye, stabilizers, and other components, giving the film its near energy and dose rate independent response (<5%).^26^ Furthermore, EBT‐3 film specifications declare a very high spatial resolution, down to 25 μm or less.^26^

For calibration, film samples were cut (2 × 3 cm^2^) and irradiated with a 6 MV photon beam with a Varian 2100C/D accelerator. Samples were placed at the build‐up in a PMMA phantom positioned on the beam axis, 20 × 20 cm^2^ field size at a source to surface distance (SSD) 100 cm. The absorbed dose was Clinac determined in the point of measurement by a PTW Roos ionization chamber, calibrated at a Standard Laboratory, according to IAEA protocol (TRS 398). Dose range was from 0 to 8 Gy. A commercial Epson Expression 10 000XL color flatbed scanner was used to digitalize the film samples in landscape orientation. The scanner had a high spatial resolution and the acquisition was performed in the red channel, in order to minimize errors all film pieces were positioned in a well‐identified area of the scanner, in landscape orientation. Films were scanned 24 h after the irradiation. Images were analyzed with Picodose X PRO software. For each dosimeter, a rectangular central area with an appropriate standard deviation was selected and the corresponding mean grey level value (GL) was obtained. The net optical density (NOD) was calculated by NOD = −log10 (GL/GL0) where GL0 was the GL for non‐irradiated film and GL was the GL corresponding to the delivered absorbed dose. Dose calibration curves, such as the function of NOD were plotted, data were fitted using a fourth‐order polynomial function. The overall uncertainty was about ±3%. Data were fitting by a four‐degree polynomial function to obtain the calibration curve of GL versus absorbed dose.

For QA verification the film was cut such that smaller films of appropriate size were obtained from each sheet of film. For scanning, these small films were placed in the center of the scanner bed and oriented on the scanner such that the original long axis of the film coincided with movement of the light source. The Epson scanner was used in transmission mode and in all EBT films were scanned in the 48‐bit, RGB mode, resolution 75 dpi, but only the red color channel image was used and saved in tagged image file format (TIFF).

Film‐based measurements were performed placing a detector between two 30 × 30 cm^2^ water solid slabs (Gammex Inc., Middleton, USA) with a physical density of 1.0431 g/cm^3^. The lower backscatter slab is 5 cm thick, while the upper slab is 2 cm thick and brings eight radiopaque fiducials on its surface for positioning tracking with onboard kilovoltage imaging.

### 2.6 Delivery quality assurance (DQA) plans

For each patient, two DQA plans were prepared by overlaying the clinical plan on the CT images of the StereoPHAN and of the water solid phantom.

All plans were calculated with the ray‐tracing algorithm or, in presence of inhomogeneities, with the Monte Carlo algorithm of the Precision TPS (Accuray Incorporated) version 2.0.1.1, using the highest dose grid resolution available, that corresponds to the CT step.

The targets were always positioned within the sensitive area of the dosimeters, in order to verify the high‐dose region delivery accuracy.

For film‐based DQA, a MU scaling factor was applied whenever the treatment plan was expected to deliver a dose outside the EBT‐3 film dose linearity range.

CK uses registration of stereoscopic x‐ray images to the planning computer tomography (CT) to locate and position the patient on the treatment couch. Patient position shifts in reference to the calibrated imaging center are tracked and corrected by the robot. Inverse treatment planning is based on sequential multi‐objective optimization, which generally results in an arrangement of several non‐isocentric non‐coplanar beams of various sizes and source‐detector‐distances (SDDs) generating complex dose distributions with steep dose gradients.^3^, ^12^

The QA necessary for robotic radiosurgery was summarized in the AAPM Task Group 135 report.^3^

### 2.7 Gamma index analysis

DQA plans were delivered on the phantoms, and the measured dose distribution were compared with those calculated by the TPS. For both GAFChromic and SRS MapCHECK measurements, the agreement was assessed using the SNC software by means of 2D gamma analysis,^27^ evaluating the percentage of points for which the gamma index was ≤1.

For both measurements methods the same evaluation criteria was chosen by using a local‐pixel‐dose‐difference of 2%, a DTA of 2 mm and a gamma pass‐rate of 90%, as suggested by the AAPM Task Group 135.^3^ For SRS MapCHECK measurements the dose threshold of the gamma function was set to 10%, while for GAFChromic measurements we decided to raise it to 30% in order to avoid points below the film dose linearity range.

### 2.8 Statistical analysis

Statistically significant differences between the results provided by SRS MapCHECK and films were found using the Wilcoxon Signed‐Rank non‐parametric test (significance *p* < 0.05).^28^

The statistical correlation between the two techniques and the association between the gamma passing rate and the target volume were studied using Spearman's rank correlation coefficient.^29^ We also used the Bland–Altman statistical method,^30^, ^31^ in order to assess if the two measurement methods can be considered interchangeable within the combined inaccuracy.

All the evaluations were performed with Matlab (The MathWorks, Inc) statistical tools.

## 3 RESULTS

### 3.1 Gamma analysis results

**FIGURE 4:**
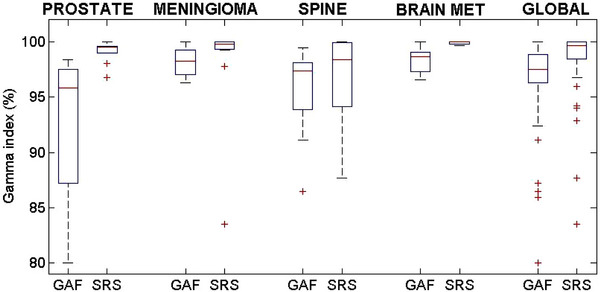
Box‐plots of the gamma analysis results obtained with EBT‐3 films and SRS MapCHECK for prostate, meningioma, spine, brain metastases, and global cases. SRS, stereotactic radiosurgery.

Gamma passing rates obtained with SRS MapCHECK and film techniques were analyzed and compared. The mean and standard deviation values of the data were chosen to define the differences between the two techniques. The results are resumed in Table 2 and Figure 4. The highest mean passing rate was observed for the SRS MapCHECK system compared to films.

**TABLE 2:** SRS MapCHECK and EBT‐3 film gamma analysis results, expressed in terms of mean ± SD (range).

	SRS MapCHECK	EBT‐3 film	*p*
Brain meningioma	99.3 ± 1.2 (95.9 ÷ 100.0)	98.3 ± 1.2 (96.3 ÷ 100.0)	1.2 ∙ 10^−1^
Spine	96.8 ± 3.7 (87.7 ÷ 100.0)	95.7 ± 3.7 (86.5 ÷ 99.5)	**3.1 ∙ 10^−2^**
Brain metastases	99.9 ± 0.1 (99.7 ÷ 100.0)	98.5 ± 1.2 (96.6 ÷ 100.0)	**7.8 ∙ 10^−3^**
Prostate	99.1 ± 1.0 (96.8 ÷ 100.0)	93.0 ± 6.3 (80.0 ÷ 98.4)	**7.8 ∙ 10^−3^**
Global cases	98.4 ± 3.3 (87.7 ÷ 100.0)	96.4 ± 4.1 (80.0 ÷ 100.0)	**8.0 ∙ 10^−6^**

*Notes*: The comparison (last column) was performed using the Wilcoxon Signed‐Rank non‐parametric test (*p* < 0.05). Statistically significant differences are highlighted in bold.

Abbreviation: SRS, stereotactic radiosurgery.

The SNC software provides a shift/rotation registration algorithm to correct for potential setup errors between measured and calculated doses. The gamma results obtained with SRS MapCHECK system reported in our work were obtained after applying this correction. Nevertheless, none of the cases had passing rates below 90% even before registration. The only exception was reported for one spine case for which the gamma result was 87.7%’’. In particular, the mean and standard deviation passing rate was 99.3% ± 1.2%, 96.8% ± 3.7%, 99.9% ± 0.1%, and 99.1% ± 1.0% for brain meningioma, spine, brain metastasis and prostate cases, respectively. The passing rate for the whole group of measurements ranged from 87.7% to 100.0% (mean 98.4% ± 3.3%).

The mean and standard deviation passing rate for film QA was 98.3% ± 1.2%, 95.7% ± 3.7%, and 98.5% ± 1.2%, 93.0% ± 6.3% for brain meningioma, spine, brain metastasis and prostate cases, respectively. The passing rate for the whole group of measurements ranged from 80.0% to 100.0% (mean 96.4% ± 4.1%).

The Wilcoxon Signed‐Rank test was performed and, for the whole group of measurements, the *p*‐value was 8.0 ∙ 10^−6^. Results <0.05 were obtained also for the single data sets, except for brain meningioma.

### 3.2 Association between SRS MapCHECK and EBT‐3 film techniques

For each treatment site, Spearman's rank correlation coefficient values obtained were *r_s_*_ _> 0.3. The test of relation significance showed that the probability p (2‐tailed) is 3.6 ∙ 10^−2^.

**FIGURE 5:**
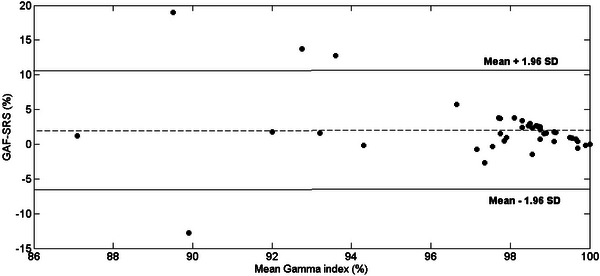
Bland–Altman test plot. On the x‐axes the mean gamma index obtained with EBT‐3 films and SRS MapCHECK for single tests is represented. On the y‐axes the difference between the gamma index obtained with EBT‐3 films and SRS MapCHECK for single tests is represented. The dashed line represents the mean difference (2.0%), while bold lines represent the (mean ± 1.96 SD) range (−6.7% ÷ 10.7%). SRS, stereotactic radiosurgery.

The Bland–Altman analysis showed that 91.3% of the gamma values differences obtained with SRS MapCHECK and films were within the (mean ± 1.96 SD) range (Figure 5).

### 3.3 Correlation between target volume and gamma passing rate

**FIGURE 6:**
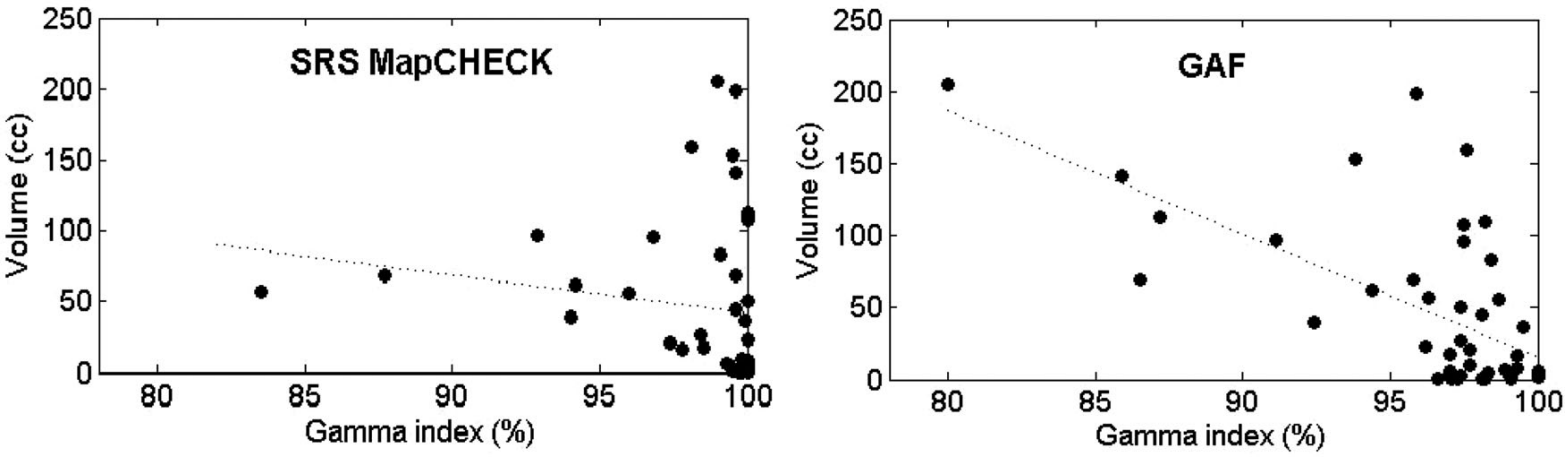
Correlation between the target volume and the gamma index passing rate for SRS MapCHECK (left) and EBT‐3 films (right). SRS, stereotactic radiosurgery.

The median tumor volume recorded was 46.0 cm^3^ (range: 0.1 ÷ 205.0 cm^3^). Figure 6 shows the correlation between the target volume and the gamma index passing rate. The results of Spearman's rank correlation analysis were *p* = 1.0 ∙ 10^−3^ and *p* = 6.0 ∙ 10^−4^ for SRS MapCHECK and EBT‐3 films, respectively.

## 4 DISCUSSION

Film dosimetry is a time and material consuming method that requires well controlled calibration. In contrast, SRS MapCHECK systems provide an efficient tool to verify SBRT treatments with minimal calibration processes, daily output corrections, and user friendly applications for QA verifications.

In order to quantify the performance of the SRS MapCHECK system, basic dosimetric tests involving targets with different sizes and prescription doses were carried out.

We evaluated the results of gamma analysis pre‐treatment verification for four different treatment sites and three collimator types, obtained with SRS MapCHECK (2%/2 mm, TH 10%) and EBT‐3 films (2%/2 mm, TH 30%).

In most cases, the Wilcoxon Signed‐Rank test highlighted a statistically significant lower gamma passing rate for film measurement with respect to the 2D array system.

Reasons can be manifold. First of all, films have a much higher spatial resolution with respect to a 2D diode array^20^ and this can lead to a greater number of points of discrepancy between the measured and calculated dose distributions. Films’ response is also susceptible to variation in handling, unlike SRS MapCHECK. Furthermore, SRS MapCHECK measurements are relative to a daily dose calibration performed directly with the CK device, whereas films have been calibrated with a standard linear accelerator. That is, with film dosimetry there could be a dependence from daily output variations that will not be taken into account. Moreover, SRS MapCHECK uses a global gamma evaluation, which is less sensitive when compared to the 2D one used for films.^32^

Previous studies compared film measurement with other QA systems and reported similar results. Chandraraj V, et al.^33^ compared EDR2 film dosimetry with IBA I'MatriXX array, PTW Seven29 array, and the Delta^4^ array dosimetry. All QA tests passed the gamma index criteria (3%/3 mm). In addition, the Bland–Altman analysis showed that all the calculated gamma values of all three QA devices were within 5% from those of the film. Bedford et al.^32^ compared Delta^4^ measurements to EDR2 and EBT film measurements in a cuboid water‐solid phantom. They reported that, on average, Delta^4^ records a higher percentage of points agreeing with the planned dose to within 3% and 3 mm than film. These results were mainly referred to the fact that Delta^4^ measurements are absolute, while films were used as relative dosimeters.

In our study, Spearman's rank correlation analysis showed that there is a significant positive relation between the SRS MapCHECK and EBT‐3 film data sets of measurements, for each treatment site. This was further supported by the Bland–Altman analysis that showed that more than 90% of the gamma values differences obtained with SRS MapCHECK and films were within the range (mean ± 1.96 SD). Therefore, our results show that the two methods can be considered interchangeable within the combined inaccuracy.

For the SRS MapCHECK system the highest mean passing rate (99.9% ± 0.1%) was observed for brain metastases treated with fixed collimator, whereas the lowest mean passing rate (96.8% ± 3.7%) was observed for spine cases treated with MLC collimator. A similar trend was recorded for EBT‐3 films, with the highest mean passing rate (98.5% ± 1.2%) observed for brain metastases and the lowest mean passing rate (93.0% ± 6.3%) observed for prostate cases treated with MLC collimator. This may be related to the fact that MLC plans usually involve targets with bigger volumes and larger average beam apertures in order to accommodate these targets. In our study, the results of Spearman's rank correlation analysis showed a statistically significant association between the gamma passing rate and the target volume: with the increase of target volumes and radiation field size, the passing rate gradually decreases.

The reason is probably related to the fact that targets with larger volumes, like spine or prostate cases, have also an irregular shape that require more complex plans with a higher modulation degree compared to fixed or iris plans. Plan complexity is generally thought to be related to the dose delivery accuracy. McNiven et al.^34^ reported no correlation between the IMRT gamma passing rates and their modulation complexity score (MCS) metric across multiple treatment sites. However, McGarry et al.^35^ suggested that lower IMRT gamma passing rates were associated with plans with higher beam complexity or lower MCS values. Furthermore, Younge et al.^36^ reported that plans with higher complexity or larger edge metric values lead to greater percentage of pixels with >10% dose errors.

The correlation between the gamma passing rate and the target volume is more prominent in EBT‐3 film tests. This, again, could be related to the higher spatial resolution of films with respect to a 2D diode array, that could cause worse gamma analysis results with increasing plan complexity.

## 5 CONCLUSIONS

The use of SRS MapCHECK has been validated for patient specific QA on CK for a variety of high‐complexity clinical plans. In some cases, the limitations of the device might be the small size of the active area and the inability to sample largest target volumes properly. In fact, the diodes are arranged in a 77 × 77 mm^2^ matrix and when field size exceeds the sensitive area of the system the gamma passing rate sharply decreases. However, SRS MapCHECK has sufficient dosimetric accuracy and spatial resolution to be a useful tool for QA and could be reliably used as a replacement for radiochromic films. The use of SRS‐MC has been characterized and validated for patient specific QA on CK for a variety of clinical plans. providing time and material saving.

## AUTHOR CONTRIBUTIONS

Erminia Infusino designed the study, analyzed the data, and wrote the manuscript with support from Anna Ianiro and Cristina Pugliatti. Stefano Luppino and Sandro Nocentini assisted with measurements. Antonella Soriani supervised the findings of this work. All authors discussed the results and contributed to the final manuscript.

## CONFLICT OF INTEREST STATEMENT

The authors declare no conflicts of interest.
